# Seminal plasma did not influence the presence of transforming growth factor-β1, interleukine-10 and interleukin-6 in porcine follicles shortly after insemination

**DOI:** 10.1186/1751-0147-55-66

**Published:** 2013-09-10

**Authors:** Jiwakanon Jatesada, Persson Elisabeth, Dalin Anne-Marie

**Affiliations:** 1 Division of Reproduction, Department of Clinical Sciences, Swedish University of Agricultural Sciences (SLU), Uppsala, Sweden; 2 Department of Anatomy, Physiology and Biochemistry, Swedish University of Agricultural Sciences (SLU), Uppsala, Sweden; 3 Present address: Group for Preventive Technology in Livestock, Khon Kaen University, Khon Kaen, Thailand

**Keywords:** Gilt, Cytokine, Ovary, Follicle, Seminal plasma

## Abstract

**Background:**

The effects of seminal plasma on the presence of the cytokines transforming growth factor (TGF)-β1, interleukin (IL)-10 and IL-6 in ovarian follicles and follicular fluid were studied shortly after insemination in gilts.

Ovaries from gilts were sampled 5–6 h after insemination with either seminal plasma (SP), fresh semen in extender (Beltsville thawing solution, BTS), spermatozoa in extender (Spz), or only BTS (control).

**Results:**

Immunohistochemical (IHC) labeling of TGF-β1, IL-10 and IL-6 was evident in the ovarian oocytes and granulosa cells independent of stage of follicular development (antral follicles). Theca interna cells were labeled to a high degree in mature follicles. No consistent differences between treatment groups could be observed for any of the cytokines.

In follicular fluid, high concentrations of TGF-β1 were found while the levels of IL-10 and IL-6 were low. There were no differences between treatment groups.

**Conclusions:**

Our results show a presence of the cytokines TGF-β1, IL-6 and IL-10 in oocytes, granulosa and theca cells, as well as in the fluid of mature follicles suggesting a role of these cytokines in intra-ovarian cell communication. However, treatment (SP, fresh semen in BTS, spermatozoa in BTS or BTS) did not influence the IHC-labeling pattern or the levels of these cytokines in follicular fluid shortly after insemination.

## Background

Boar seminal plasma (SP) contains many factors essential for early reproductive processes. For example, following insemination, seminal estrogens stimulate uterine myometrial contractions [1] which facilitate sperm transport to the UTJ (utero tubal junction), and various SP proteins from boars contribute to sperm capacitation [2,3], formation of the oviductal sperm reservoir [3,4] and gamete interaction [5].

It has been shown previously that natural mating of sows [6] and insemination of gilts with SP [7,8] early in oestrus have an effect on the onset of ovulation. The interval between oestrus and ovulation was reduced on average 8 h by SP [9]. Waberski *et al*. [10] suggested that cytokines in the uterine lymph, when induced by semen, may undergo counter-current transfer to the ovary and thereby accelerate the final maturation of pre-ovulatory, mature follicles. Supporting this hypothesis, a local transfer of ^125^I-labelled TNF-α from inflamed porcine uterine tissue to ovarian parenchyma was demonstrated by Kucharski *et al*. [11]. Furthermore, O’Leary *et al*. [12] found that 34 h after insemination with SP in hCG-treated gilts, the number of leukocytes (CD45 and swine leukocyte class II) in ovarian tissue was significantly higher than in control gilts, suggesting that uterine exposure to SP influences the presence of immune cells in the ovary.

The TGF-β superfamily is suggested to be an important intraovarian regulator of follicular development, i.e. the bi-directional communication between oocyte and granulosa cells, as well as between granulosa cells and theca cells (see review by [13]). With IHC, Sriperumbudur *et al.*[14] showed cells positively labeled for the cytokines TGF-β (β1 and β2) in preovulatory (granulosa cells) as well as in postovulatory follicles (luteinizing cells). The expression of porcine IL-6 mRNA has been demonstrated in the granulosa cells of normally developed and atretic follicles [15] as well as in the corpus luteum at all luteal stages [16].

We have previously studied the effects of SP on the cytokines TGF-β1, IL-6 and IL-10 in different parts of the oviduct [17] and endometrium [18] both shortly after insemination (5–6 h) and later (35–40 h). The results showed that at 35–40 h after infusion, not only SP but also semen extender (BTS) and spermatozoa in BTS, up-regulated TGF-β1 mRNA expression compared with the control (catheter insertion) in the oviduct (isthmus and infundibulum). In the endometrium, however, a significant down-regulation of TGF-β1 mRNA was observed after insemination with BTS and spermatozoa in BTS but not with SP compared with the control. The mRNA expression of IL-10 and IL-6 in both the oviduct and the endometrium were significantly lower than for TGF-β1. IHC-labeling of TGF-β1, IL-10 and IL-6, was evident, especially in the epithelial cells of both the oviduct [17] and the endometrium [18].

SP contains the cytokines TGF-β1 [17,19,20] and IL-10 as well as IL-6 [17,20]. These cytokines have also been reported in ovarian tissue [14-16] and it is therefore of interest to study the effects of SP, compared with insemination components, on cytokines in mature follicles.

The aim of the present study was therefore to examine the effects of SP, spermatozoa in extender (BTS), fresh semen in extender and extender only (control) on the presence of the cytokines IL-6, IL-10 and TGF-β1 in ovarian follicles and follicular fluid shortly after insemination in gilts.

## Material and methods

The research plan was approved by the Ethical Committee for Experimentation with Animals, Uppsala, Sweden. Sixteen crossbred gilts (Swedish Landrace × Swedish Yorkshire) were used in the present study. The animals and their management have been described earlier, since the animals were used in another study on cytokines in the oviduct (see [17]; the first experiment). After natural puberty (no hormonal induction was used), the gilts were checked twice daily for the onset of pro-oestrus, where after control of the standing reflex in the presence of a boar was performed every 4 h. The gilts were inseminated once in their second or third oestrus at 15–20 h after the first signs of standing reflex (about 20–15 h before expected ovulation) with 100 ml of either seminal plasma (n = 4), spermatozoa in extender [Beltsville thawing solution, BTS [21], n = 4], fresh semen in extender (n = 4) or extender (BTS) alone as control (n = 4).

### Semen collection and preparation

For details, see [17]. Seminal plasma was prepared in advance from semen that had been collected from four boars of known fertility, using the gloved-hand technique. Semen was pooled and centrifuged, and the SP was separated from the sperm layer and centrifuged again twice, to remove any remaining spermatozoa. The recovered SP was aliquotted (100 mL) and stored at −20°C until used for insemination. For insemination with fresh semen, an ejaculate was collected from one boar at the time when the gilts started to show the standing reflex. The sperm motility was checked (> 70%) and the sperm concentration was calculated. The semen was diluted with extender (BTS) [21] to give a dose containing 5 × 10^9^ spermatozoa in a total volume of 100 mL.

For insemination with spermatozoa without seminal plasma, spermatozoa were isolated from SP using the single layer centrifugation technique, SLC [22,23]. Briefly, a layer of semen extended in BTS was carefully layered on top of colloid solution (Androcoll™-P; SLU) and centrifuged. The sperm pellet generated was carefully transferred to a new tube containing BTS and washed by centrifugation before BTS was added to generate a dose of 100 ml containing 5 × 10^9^ spermatozoa for insemination.

### Examination of the reproductive organs and tissue collection

The gilts were slaughtered (euthanized) 5–6 h after insemination. Immediately after slaughter, the genital organs were collected and examined for normality. The numbers of mature follicles (9–10 mm in diameter) were counted. Follicular fluid was sampled from all the mature follicles of both ovaries using a sterile needle and syringe, put into cryo-tubes, plunged into liquid nitrogen and stored at −80°C until analyzed for cytokine concentrations. Thereafter, samples of ovarian tissue were immersion-fixed in 2% paraformaldehyde and then embedded in paraffin blocks followed by sectioning for immunohistochemical labelling.

### Cytokine determination

Samples of follicular fluid from individual gilts (pooled sample from all mature follicles) were analyzed with commercially available ELISA kits for detection of the porcine cytokines IL-6, IL-10 and TGF-β1 (P6000, IL-6; P1000, IL-10; and MB10B, TGF-β1; Quantikine Porcine Immunoassays, R&D System Europe Limited, Abingdon, UK). The minimum limits of detection were as follows: IL-6 ≤ 10 pg/ml, IL-10 ≤ 3.5 pg/ml and TGF-β1 ≤ 4.6 pg/ml. Optical density readings were performed at 450 nm and corrected at 540 nm using a Thermal Lab systems Multiskan EX ELISA reader.

### Blood collection and hormone assays

To confirm hormonal status, a blood sample was collected from the jugular vein one hour prior to slaughter. Blood samples were analyzed by radioimmunoassays for plasma oestradiol-17β (E_2_) and progesterone (P4) with no significant differences between the treatment groups; see Table 1[17].

**Table 1:** Plasma levels of oestradiol-17β and progesterone (samples taken 1 h before slaughter) in the experimental groups of gilts (mean ± SD)

**Experimental group**	**Progesterone (nmol/l)**	**Oestradiol-17β (pmol/l)**
Seminal plasma	1.0 ±0.7	20.7 ±10.6
Spermatozoa in BTS	1.8 ±1.6	12.0 ±3.6
Fresh semen in BTS	1.6 ±2.0	12.3 ±5.1
BTS*	2.3 ±1.9	18.8 ±19.6

*BTS = Beltsville thawing solution.

### Immunohistochemical labelling

For detection and localization of cytokines (IL-6, IL-10 and TGF-β1) in ovarian follicles, a standard avidin-biotin immunoperoxidase technique was used. The immunohistochemical protocol has been described previously [17]. Briefly, heat-induced (microwave) antigen retrieval was applied to the deparaffinized sections and an avidin-biotin complex (ABC) kit (Vectastain® ABC kit for Mouse IgG, PK-6102 or Rabbit IgG, PK-6101, Vector Laboratories, Inc., Burlingame, CA, USA) was used. Endogenous peroxidase activity was blocked by hydrogen peroxidase in methanol. Non-specific protein binding was diminished using either horse serum (for the monoclonal antibodies used) or goat serum (for the polyclonal antibody used). The sections were incubated overnight with the primary antibodies or isotype matched controls (Table 2). In the final step, 3,3’-diaminobenzidine (DAB, Dakopatts AB, Älvsjö, Sweden) was added as chromogen and all sections were counterstained with Mayer’s Haematoxylin. For each cytokine, one reference section was run in every batch of IHC as an inter-assay control.

**Table 2:** Antibodies used for cytokine detection

**Specificity**	**Isotype**	**Clone number**	**Source**
IL-6	Mouse IgG_1_	MAB686	R&D System Inc., Minneapolis, MN, USA
IL-10	Mouse IgG_2B_	MAB6932	R&D System Inc., Minneapolis, MN, USA
TGF-β1	Rabbit IgG	SC-146	Santa Cruz Biotechnology Inc., Santa Cruz, CA, USA
Non-immune serum	Mouse IgG_1_ (IL-6 control)	MAB002	R&D System Inc., Minneapolis, MN, USA
Non-immune serum	Mouse IgG_2B_ (IL-10 control)	MAB0042	R&D System Inc., Minneapolis, MN, USA
Non-immune serum	Rabbit IgG (TGF-β1 control)	011-000-003	Jackson ImmunoResearch Europe Ltd., Suffolk, UK

Photographs of mature follicular walls taken in a Nikon-FXA photomicroscope (Nikon Corporation, Tokyo, Japan), with objective × 40 and eyepieces × 10, were examined for cytokine quantification. The IHC-labeling was evaluated on the photographs by two persons. A scoring system for positive labeling including cell proportion and intensity (0 = none, 1 = low, 2 = medium, and 3 = high level) was used.

### Statistical analyses

The concentrations of the cytokines TGF-β1, IL-10 and IL-6 in follicular fluid were statistically analyzed using the SAS statistical package (version 9.1.3, SAS Institute, Inc., 2002–2003, Cary, NC, USA). Normal distribution of residuals from the statistical models was tested using the UNIVARIATE procedure option NORMAL. Differences in mean of each cytokine concentration between treatment groups (four groups: SP, spermatozoa, fresh semen and extender) were tested using analysis of variance (Proc GLM). Bonferroni t-test was used to compare least-square mean values between experimental groups when an overall significance for the effect was found. A P-value ≤ 0.05 was considered statistically significant.

## Results

### Clinical observations and macroscopic findings

The gilts were inseminated at 16.9 ± 2.3 h (mean ± SD; range 15–20) after the start of the standing reflex. The ovaries were normally developed, weighing 6.4 ± 1.9 g. Thirteen of the 16 gilts had mature follicles [mean number 15.7 ± 1.97 (range 13–19)] and regressed corpora lutea. Three gilts were excluded from the study due to having newly ovulated follicles (two gilts, one in the fresh semen group and another in the BTS group) or follicles under ovulation (one gilt; in the seminal plasma group).

### Immunohistochemistry

**Figure 1:**
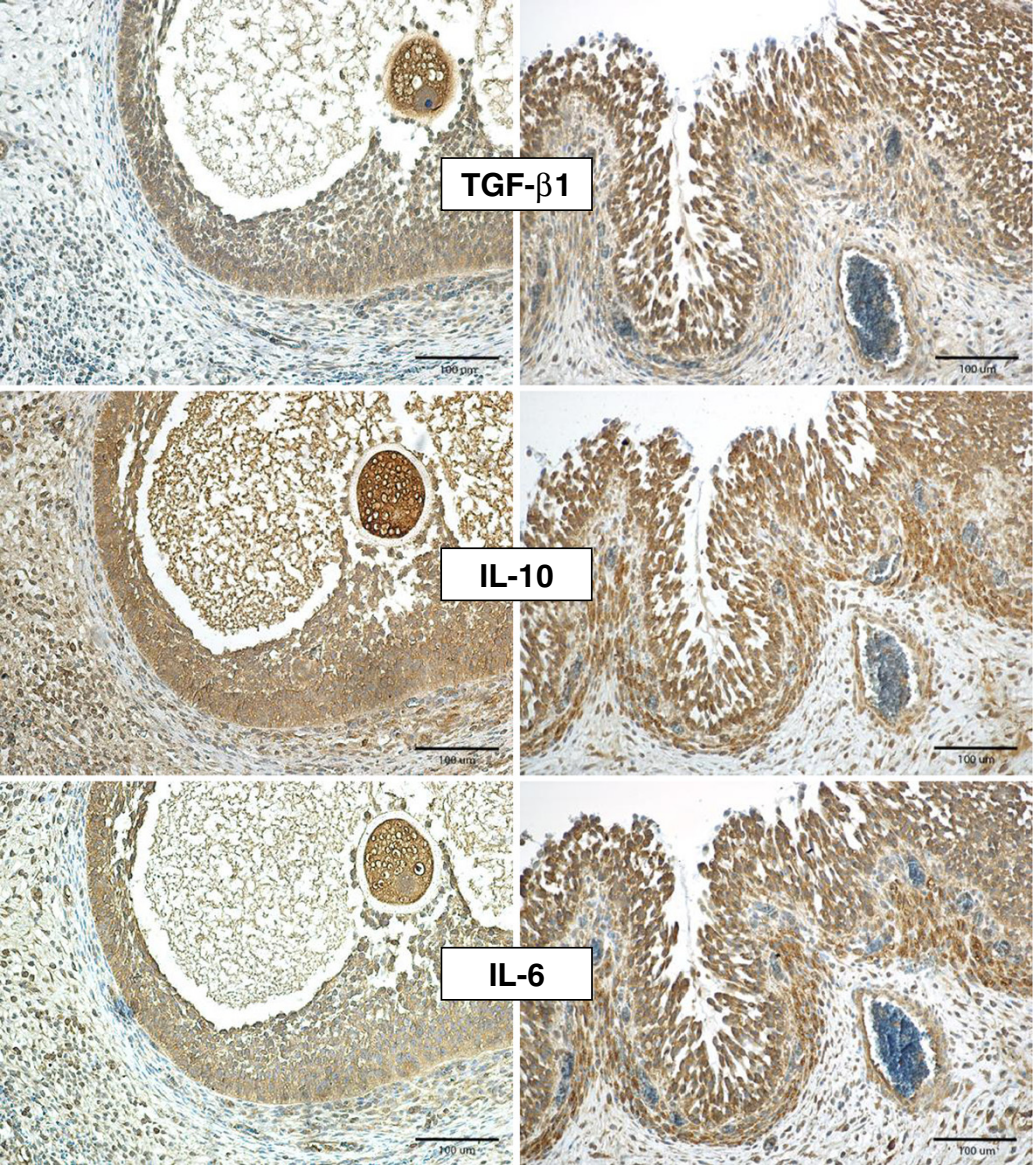
**Immunohistochemical labeling of TGF-β1, IL-10 and IL-6 in the porcine ovary collected 5–6 h after insemination.** Representative photo from a gilt in the group inseminated with seminal plasma. Two stages of antral follicular development (mature and at earlier stage of development) are shown.

**Figure 2:**
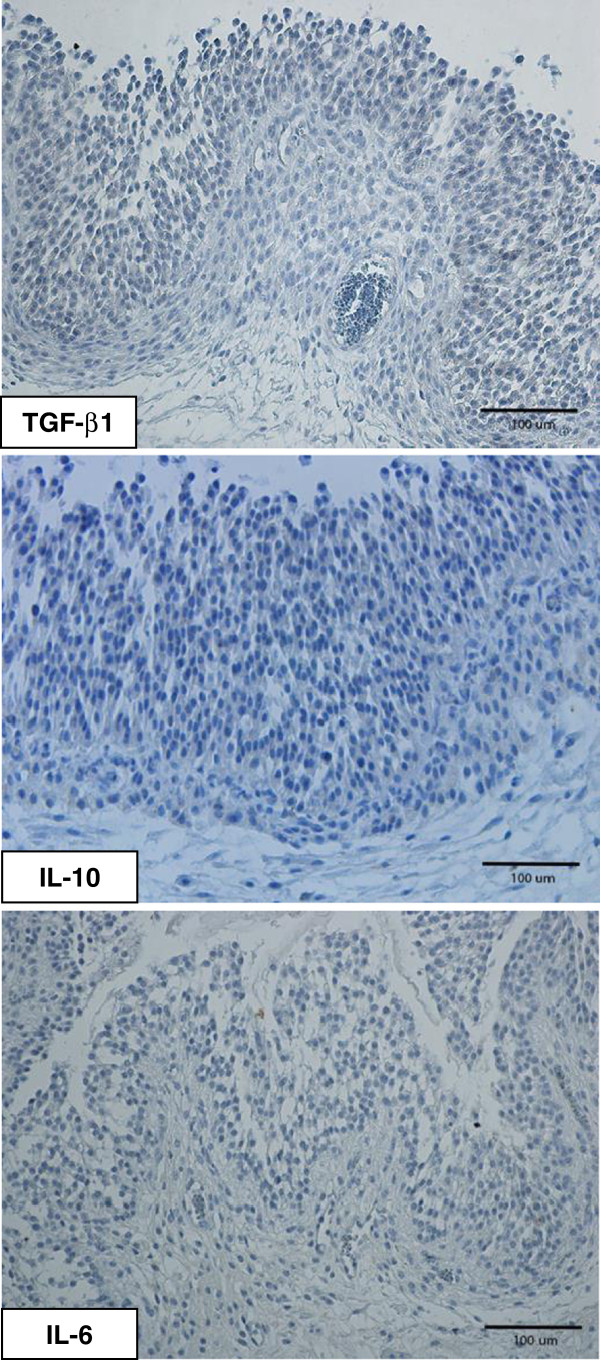
**Immunohistochemical labeling of TGF-β1, IL-10 and IL-6 in the porcine ovary collected 5–6 h after insemination.** Negative controls.

**Figure 3:**
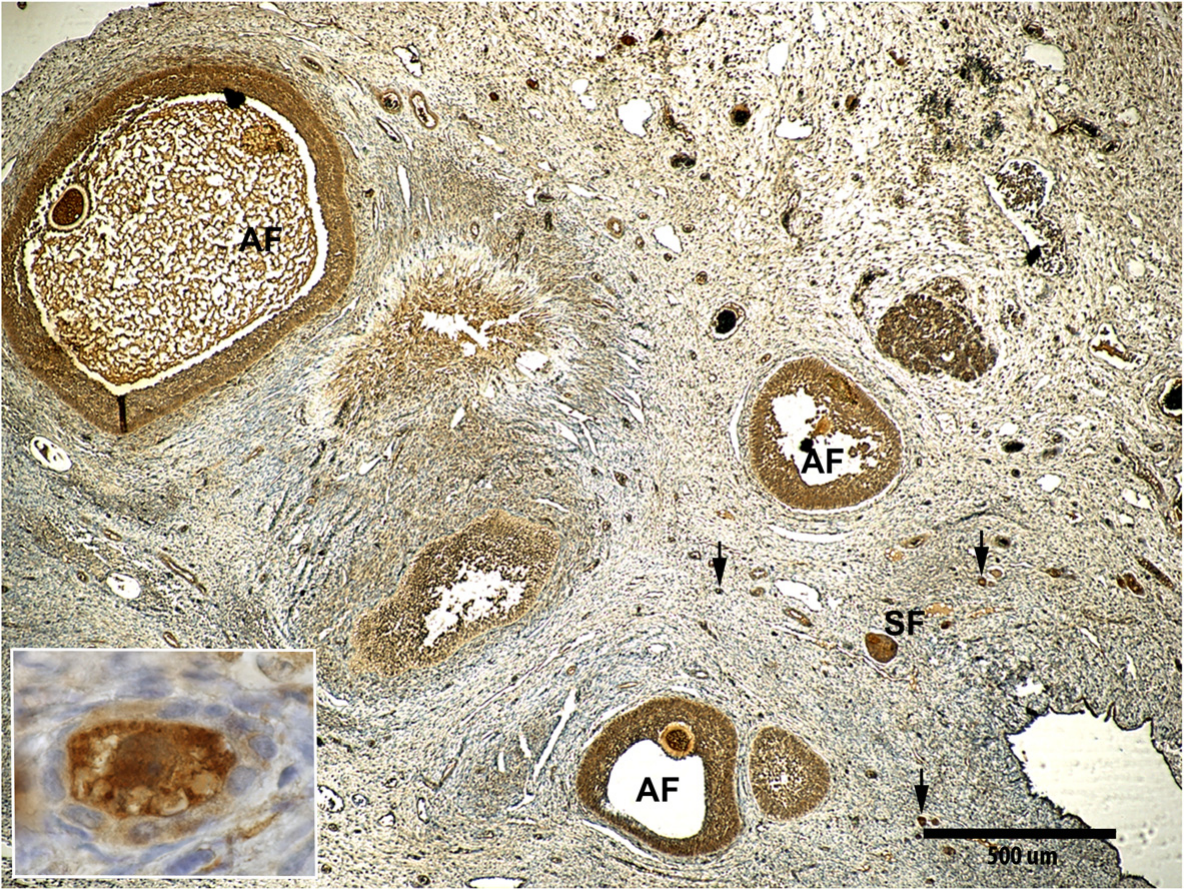
**Immunohistochemical labeling of TGF-β1 in a porcine ovary collected 5–6 h after insemination.** Different follicular stages (antral follicle = AF, secondary follicle = SF and primary follicle = arrow) are shown and a magnified primary follicle is inserted. Representative photo from a gilt inseminated with seminal plasma.

In Figure 1, representative immunolabeling patterns of TGF-β1, IL-10 and IL-6 in the walls of antral follicles, mature and in earlier stages of development, are shown for gilts from the seminal plasma group. Figure 2 shows the negative controls. The patterns of labeling for TGF-β1, IL-10 and IL-6 were similar. Generally, positive staining was present in the oocytes and the granulosa cell layers of antral follicles independent of stage of follicular development (Figures 1 and 3). The staining intensity of oocytes was consistently strong and independent of developmental stage of antral follicle (data not shown). In primordial follicles, labeling of some follicular epithelial cells was also observed, while higher proportions of granulosa cells were labeled in primary and secondary follicles. In fully mature follicles, all theca interna cells seemed to be positively stained for TGF-β1, IL-10 and IL-6 (Figure 1) and scattered positive cells were found also in theca externa (Figure 1). At earlier development stages of antral follicles, a higher proportion of theca interna cells were stained in the area close to the cumulus oophorus than in the other areas (Figure 1). The scoring results of positive labelling (both granulosa and theca cell layers) for TGF-β1, IL-10 and IL-6 in all treatment groups are shown in Table 3. No significant difference was found between treatment groups, although less variation in scoring was found for TGF-β1 (range 2–3) than for IL-10 and IL-6 (range 1–3).

**Table 3:** Scoring of TGF-β1, IL-10 and IL-6 based on immunohistochemical labeling of mature follicles (both granular cell and theca cell layers evaluated) from different treatment groups [seminal plasma (SP), n = 3; spermatozoa in BTS (Sperm), n = 4; fresh semen in BTS (FS), n = 3 and Beltsville thawing solution (BTS), n = 3], sampled 5–6 h after insemination

**Treatment group**	**TGF-β1**	**IL-10**	**IL-6**
SP	2, 2, 3	1, 1, 3	1, 3, 3
Sperm	2^a^, 3, 3, 3	2, 2, 3, 3	1, 2, 3, 3
FS	2, 3, 3^a^	1, 2^b^, 2^b^	2, 3, 3
BTS	2, 2, 2	1, 2, 3	2, 3, 3

^a^ = higher intensity in granular cell layer than theca cell layer.

^b^ = higher intensity in theca cell layer than granular cell layer.

### Cytokines in follicular fluid

A high concentration of TGF-β1 was found in all follicular fluid samples (Table 4). However, the variation between samples within group was high and possible differences between treatments were, therefore, not found. The mean concentrations of IL-10 and IL-6 were much lower than the concentration of TGF-β1 (Table 4). Detectable levels of IL-10 were found in 9 out of 13 samples analyzed and the concentrations did not differ between treatment groups due to high variation. Only two samples contained detectable levels of IL-6. The concentrations of the cytokines in follicular fluid did not correlate to the immunohistochemical scoring patterns for the respective cytokine.

**Table 4:** TGF-β1, IL-10 and IL-6 concentrations (mean ± SD, pg/ml) in follicular fluid from different treatment groups [seminal plasma (SP), n = 3; spermatozoa in BTS (Sperm), n = 4; fresh semen in BTS (FS), n = 3 and Beltsville thawing solution (BTS), n = 3], sampled 5–6 h after insemination

**Treatment group**	**TGF-β1**	**IL-10**	**IL-6**
Seminal plasma	801.5 ±315.1	11.9 ±9.3	22.8^a^
Spermatozoa in BTS	1403.7 ±626.9	11.58^a^	35.0^a^
Fresh semen in BTS	1646.1 ±1903.7	51.6 ±47.6^b^	ND
BTS	1382.7 ±848.3	9.5 ±6.0^b^	ND

*BTS* = Beltsville thawing solution, ^a^ = from only one positive sample, ^b^ = from two positive samples, *ND* = not detectable.

## Discussion

The aim of the present study was to examine the effects of SP, spermatozoa in extender (BTS), fresh semen in extender and extender only (control) on the presence of the cytokines IL-6, IL-10 and TGF-β1 in ovarian follicles and follicular fluid shortly after insemination in gilts. The present study showed that the concentration of TGF-β1 was much higher than the concentrations of IL-10 and IL-6 in the fluid from mature porcine follicles. The patterns of TGF-β1, IL-10 and IL-6 presence as shown by IHC-labeling of the follicles were similar; the scores of the labeling varied less for TGF-β1 than for IL-10 and IL-6.

The presence of TGF-β1 and IL-10 in porcine follicular fluid has, to our knowledge, not been reported previously. The high level of TGF-β1 (mean levels about 800–1650 pg/mL) is similar to levels reported in bovine follicular fluid [24] and the low concentration of IL-10 (range of 4–85 pg/ml) is comparable to the results found by Geva *et al.*[25] in women (range of 10–350 pg/mL). The detected concentration of IL-6 (23–35 pg/mL) agrees with the low level (around100 pg/mL) found earlier from normally developed follicles in ovaries from pigs [15] and women [26].

Apparent IHC-labeling has previously been demonstrated for TGF-β1 in porcine granulosa cells of preovulatory follicles [14]. Positive IHC- labeling of the cytokines investigated in the present study (TGF-β1, IL-10 and IL-6) was found primarily in the granulosa cells of all antral follicles, independent of developmental stage, as well as in the oocytes. In mature follicles, theca interna cells were labeled to a higher degree than in follicles less developed. May *et al.*[27] found TGF-β1 mRNA expression by in situ hybridization in both granulosa and theca cells in the follicles of prepubertal gilts, although their results on cell cultures suggested that only the theca cells translated and secreted this cytokine. They also showed, from culture of intact hemi-follicle linings, that the secretion of TGF-β1 increased with increasing follicular size. In the present study, the labeling of the different cytokines in the cells of mature follicles seemed to be similar while the cytokines in follicular fluid varied with the TGF-β1 levels being high and the IL-10 and IL-6 levels low or undetectable. The results on IL-6 were notable, since only two gilts had detectable concentrations of IL-6 in follicular fluid whereas their follicular walls (granulosa and theca cells) were positively labeled for IL-6, indicating that that there is no correlation between IHC presence of the cytokine in granulosa cells and content of the same cytokine in follicular fluid.

As reviewed by Knight and Glister [28], studies in several species, especially in rodents, indicate that receptors, signaling intermediaries and binding proteins associated with the TGF-β superfamily, are expressed by oocytes and ovarian somatic cells in a developmental stage-related manner. The present study showed that oocytes, independent of follicular development, demonstrated positive labeling not only for TGF-β1 but also for IL-10 and IL-6, suggesting that these two cytokines may also play a role in the bi-directional communication between oocytes and granulosa cells.

Waberski *et al.*[7-9] found that seminal plasma (SP) stimulated induction of ovulation in inseminated gilts early during oestrus (immediately after the start of the standing reflex). In the present study, comparing inseminated fluids with BTS used as control, no differences were found between the groups inseminated with seminal plasma, spermatozoa in BTS, and fresh semen in BTS, either in IHC-labeling of the follicular cells, or in levels of IL-6, IL-10 and TGF-β1 in follicular fluid. Several possible reasons may explain the non-appearance of SP-effect in the present study. First, the cytokines studied may not be involved in the final maturation process of follicles leading to ovulation even if e.g. the TGF-superfamily, including TGF-β1, is suggested to be important for intra ovarian communication between cells see [28]. Furthermore, the interval between treatment and collection may have been too short (5–6 h) or the time for collection of ovarian tissue may have been too close to ovulation to detect any differences based on treatment. The latter suggestion is strengthened by the earlier study of Waberski *et al.*[9], who found that SP did not shorten the interval to ovulation if the treatment was done too close to ovulation.

The presence of TGF-β1, Il-10 and Il-6 in porcine follicles indicate that these cytokines have a role in the process of follicular and oocyte development and therefore need to be further investigated.

## Conclusion

Our results demonstrate a presence of the cytokines TGF-β1, IL-6 and IL-10 in oocytes, granulosa and theca cells, as well as in the fluid of mature porcine ovarian follicles, suggesting a role of these cytokines in intra-ovarian cell communication. However, shortly after insemination, no significant differences were found by the treatments (SP, fresh semen in BTS, spermatozoa in BTS or BTS) on IHC-labeling patterns or levels of these cytokines in follicular fluid.

## Competing interests

The authors declare that they have no competing interests.

## Authors’ contributions

JJ participated in the planning of the study and in the sampling, did all the IHC as well as the statistical analyses and drafting of the manuscript. AMD was responsible for the study, participated in the sampling, discussed the interpretation of the results and contributed to the writing of the manuscript. EP participated in the design of the study, discussed the results and helped to revise the manuscript. All authors have read and approved the final version of the manuscript.
